# *Leishmania donovani* Utilize Sialic Acids for Binding and Phagocytosis in the Macrophages through Selective Utilization of Siglecs and Impair the Innate Immune Arm

**DOI:** 10.1371/journal.pntd.0004904

**Published:** 2016-08-05

**Authors:** Saptarshi Roy, Chitra Mandal

**Affiliations:** Cancer Biology and Inflammatory Disorder Division, CSIR-Indian Institute of Chemical Biology, Kolkata, India; University of Notre Dame, UNITED STATES

## Abstract

**Background:**

*Leishmania donovani*, belonging to a unicellular protozoan parasite, display the differential level of linkage-specific sialic acids on their surface. Sialic acids binding immunoglobulin-like lectins (siglecs) are a class of membrane-bound receptors present in the haematopoetic cell lineages interact with the linkage-specific sialic acids. Here we aimed to explore the utilization of sialic acids by *Leishmania donovani* for siglec-mediated binding, phagocytosis, modulation of innate immune response and signaling pathways for establishment of successful infection in the host.

**Methodology/Principle Findings:**

We have found enhanced binding of high sialic acids containing virulent strains (AG83^+Sias^) with siglec-1 and siglec-5 present on macrophages compared to sialidase treated AG83^+Sias^ (AG83^-Sias^) and low sialic acids-containing avirulent strain (UR6) by flow cytometry. This specific receptor-ligand interaction between sialic acids and siglecs were further confirmed by confocal microscopy. Sialic acids-siglec-1-mediated interaction of AG83^+Sias^ with macrophages induced enhanced phagocytosis. Additionally, sialic acids-siglec-5 interaction demonstrated reduced ROS, NO generation and Th2 dominant cytokine response upon infection with AG83^+Sias^ in contrast to AG83^-Sias^ and UR6. Sialic acids-siglecs binding also facilitated multiplication of intracellular amastigotes. Moreover, AG83^+Sias^ induced sialic acids-siglec-5-mediated upregulation of host phosphatase SHP-1. Such sialic acids-siglec interaction was responsible for further downregulation of MAPKs (p38, ERK and JNK) and PI3K/Akt pathways followed by the reduced translocation of p65 subunit of NF-κβ to the nucleus from cytosol in the downstream signaling pathways. This sequence of events was reversed in AG83^-Sias^ and UR6-infected macrophages. Besides, siglec-knockdown macrophages also showed the reversal of AG83^+Sias^ infection-induced effector functions and downstream signaling events.

**Conclusions/Significances:**

Taken together, this study demonstrated that virulent parasite (AG83^+Sias^) establish a unique sialic acids-mediated binding and subsequent phagocytosis in the host cell through the selective exploitation of siglec-1. Additionally, sialic acids-siglec-5 interaction altered the downstream signaling pathways which contributed impairment of immune effector functions of macrophages. To the best of our knowledge, this is a comprehensive report describing sialic acids-siglec interactions and their role in facilitating uptake of the virulent parasite within the host.

## Author Summary

Sialic acids are nine carbon sugars present on terminal cell surface glycoproteins and glycolipids. Siglec is a membrane receptor that belongs to an immunoglobulin super family present in almost all the haematopoetic cell lineages. There are 14 different types of siglecs present on human immune cells that take an active part in balancing the magnitude of immunological reactions. In general, these siglecs bind with sialic acids and negatively regulate the immune response. *Leishmania* contains sialic acids on its surface. Virulent parasites utilize this sugar to bind with macrophages through siglec-1 and siglec-5 compared to low sialic acids containing avirulent parasites. Such sialic acids-siglec-mediated interactions exhibited a suppressed host immune response which helped them to establish successful infection compared to desialylated virulent and avirulent parasites, as well as, siglec-depleted macrophages. Interestingly, interaction between sialic acids and siglec-1 induced enhanced phagocytosis, while sialic acids-siglec-5 interaction upregulated the phosphatase SHP-1. This interaction with the virulent strain exhibited deactivation of various downstream signaling pathways and ultimately controlled translocation of a functional component of transcription factor NF-κβ for regulation of cytokines and other effector molecules in infected macrophages. Thus, the interaction between the parasite and the host cells through sialic acids-siglec binding is clearly a newly identified mechanism by which parasites can establish successful infection by subverting the host’s innate immune response.

## Introduction

Visceral leishmaniasis (VL) caused by the species *Leishmania donovani*, is the most severe form of leishmaniasis and is endemic in the region of Indian subcontinent. Due to its digenic nature, parasites multiply as an amastigote form within the phagolysosome of macrophages in the liver and spleen of the host [1]. Thus, entry and survival inside the macrophage is an imperative factor for the fulfillment of pathogenesis.

Sialic acids (Sias) are acidic sugars containing nine carbon backbones predominantly present in the terminal residue of the cell surface as well as secreted glycoproteins and glycolipids [2]. Sias participate in an array of cellular functions such as ligand for sialic acids binding immunoglobulins like lectins (siglecs), shielding element for many receptors, negatively charged barrier for various cells even pathogens, regulation of immune cell activation and leukocyte extravasations [3]. An array of human pathogens such as *Haemophilus influenza*, *Pseudomonas aeruginosa*, *Escherichia coli*, *Neisseria meningitides*, *Campylobacter jejuni and Trypanosoma cruzi* either acquire or synthesize Sias for successful infection by dampening the host immune system [4, 5].

Siglecs come under the group of immunoglobulin-type (I-type) lectins present mainly on haemotopoetic cell lineages with a vast structural diversity in recognition of their ligands. Fourteen human and nine murine siglecs have been reported so far [6]. Siglec-3/CD33 group is the major group of siglecs with a high degree of interspecies sequence homology. Most of the siglecs contain immunoreceptor tyrosine-based inhibitory motif (ITIM) in the cytoplasmic domain. One of the exceptions is siglec-1 (CD169/sialoadhesin), which has an extended extracellular structure with no ITIM bearing motif in cytosolic region [7]. ITIM motif gets activated upon ligand binding to the siglec and recruits SH2-domain containing protein tyrosine phosphatase 1/2 (SHP-1/2) to carry out various signaling pathways [8].

We have previously reported the presence of sialoglycans, especially 9-*O*-acetylated form of Sias both on promastigote and amastigote of *Leishmania donovani* [9–16]. Although the function of 9-*O*-acetylated Sias in entry and virulence of parasite was reported earlier, no systemic investigation on the role of sialic acids on dampening of host innate immune response by modulation of signaling pathways has been addressed properly. Macrophages are the safe house of this parasite. Sialic acids recognizing receptors (siglec-1 and siglec-5) are present in this immune cell. Hence the detailed study of the involvement of these siglecs in binding, phagocytosis, modulation of SHP-1 and downstream signaling pathways during *Leishmania* infection became worthwhile.

Our current findings established the involvement of sialic acids of *L*. *donovani* in siglec-1 mediated binding and phagocytosis in macrophages. Furthermore, we demonstrated altered downstream signaling pathways leading to suppression of effector functions of innate immune response specifically due to this Sias-siglec-5 interaction. Such in-depth comparative study between high Sias containing virulent strain AG83 (AG83^+Sias^) and sialidase treated AG83 (AG83^-Sias^) along with low Sias containing avirulent strain UR6 conclusively ascertain the important role of sialic acids in siglec-mediated interaction and altered immune regulation for establishment of successful infection.

## Materials and Methods

### Ethics statement

All the animal-related experiments were performed according to the National Regulatory Guidelines issued by Committee for the Purpose of Control And Supervision of Experiments on Animals (CPCSEA), Ministry of Environment and Forest, Govt. of India. Use of Balb/c mice was approved by the Institutional Animal Ethics Committee of CSIR-Indian Institute of Chemical Biology, Kolkata, India with license number 147/1999/CPCSEA. Animals were housed under standard condition such as temperature (25 ± 1°C), relative humidity (55 ± 10%) and 12 hr/12 hr light/dark cycles and fed with standard diet.

### Materials

Fluorescein isothiocyanate (FITC), bovine serum albumin (BSA), 4',6-diamidino-2-phenylindole (DAPI) and Giemsa stain were obtained from Sigma (St. Louis, MO). Sialidase was from Roche Applied Science (Mannheim, Germany); Mounting medium was from Amersham Biosciences (Uppsala, Sweden); *Sambucus Nigra* lectin (SNA) and *Maackia amurensis* lectin (MAL) were from Vector Labs; 2'7'- dichloro dihydro fluorescein diacetate acetyl ester (H_2_DCFDA) was from Molecular probes (OR, USA); siglec-E siRNA was from santa cruz Biotechnology and DyNAmo ColorFlash SYBR Green qPCR kit was from Thermo Scientific (USA). All the cytokine ELISA kits, PE-rat anti-mouse CD14 and SHP-1 antibody were obtained from BD pharmingen and BD Biosciences (USA). Anti-Siglec-E antibody was from R&D systems (MN, USA); RNeasy Mini Kit was from Qiagen (Limburg, Netherlands); Reverse Transcriptase Kit was from Promega (WI, USA) and PCR reagent system was from Invitrogen (CA, USA). All other antibodies were from Cell Signaling Technologies (MA, USA), unless indicated otherwise.

### Parasite culture

*L*. *donovani* sodium antimony gluconate (SAG) sensitive strain AG83 (MHOM/IN/1983/AG83) was originally isolated from an Indian patient with Kala-azar at Calcutta National Medical College, Kolkata. Recently this has been deposited to American type culture collection (ATCC) by Dr. Nahid Ali from this Institute. Parasite was maintained in M-199 medium supplemented with 10% heat inactivated fetal calf serum (FCS,v/v) and gentamycin sulphate (200 μg/ml) at 22°C. These parasites are designated as parasite^+Sias^. To ensure the virulence, parasites were routinely passage through hamster. Additionally, SAG sensitive Dd8 (MHOM/IN/80/Dd8) and untyped 2001; SAG resistant GE1 (MHOM/IN/90/GE1) and untyped 39 along with an avirulent strain UR6 (MHOM/IN/78/UR6) were obtained from CSIR-Central Drug Research Institute (CDRI), Lucknow and maintained similarly [17, 18]. UR6 failed to establish *in vivo* infection when injected through intracardiac route in hamster. Macrophages are capable of producing superoxide even upon UR6 infection and thereby give protection [18–20].

### Cell culture

Murine macrophage cell line J774A.1, Human monocytic cell line THP-1 and Chinese hamster ovary (CHO)-wild type (WT) cell line were obtained from ATCC. J774A.1 was cultured in IMDM supplemented with 10% heat-inactivated FCS and antibiotic antimycotic solutions at 37°C with 5% CO_2_ [21]. THP-1 and CHO-WT cells were maintained similarly in RPMI medium. Different siglec-transfected CHO cells namely CHO-siglec-1, CHO-siglec-3, CHO-siglec-5, CHO-siglec-7, CHO-siglec-9, and CHO-siglec-10 were routinely cultured in F-12 medium. Many of these siglecs are reported to be present on macrophages and monocytes.

### Dot blot

Dot blot was performed as described previously with some modifications [22]. Briefly, log phase AG83^+Sias^ promastigotes (1×10^7^) were suspended in phosphate buffer saline (PBS) and blotted onto the nitrocellulose membrane. After air drying, blots were blocked in PBS-BSA (3%), overlaid with soluble human siglec-Fc chimeras, washed and incubated with horseradish peroxidase (HRP)- protein-A for 2 hr at 25°C. After washing, blots were developed by di-amino benzidine (DAB) in H_2_O_2_ solution and quantified by densitometric analysis using Master Totallab Software, version 1.11.

### Sialidase treatment and FITC labeling of parasites

*L*. *donovani* promastigotes grown in FCS-containing medium is designated as parasite^+Sias^. They were treated with sialidase as described previously with some modifications [9]. Briefly, promastigotes were initially incubated with 9-*O*-acetyl haemagglutinin esterase of Influenza C virus (100 U/ml, 100 μl/tube) for 1 hr at 37°C to remove *O*-acetyl group. Subsequently, esterase-treated cells were washed and incubated with *Arthrobacter ureafaciens* sialidase (100 mU/ml, 10 mU/100 μl/tube) for 30 min for removal of Sias from the surface. Parasites were washed and designated as parasite^-Sias^. Parasite^+Sias^ and parasite^-Sias^ were labeled with FITC as stated earlier [21]. Log phase promastigotes were suspended in 0.1% FITC containing carbonate buffer (pH 8.0) and incubated for 1 hr at 22°C [19]. Parasites were washed and suspended in medium supplemented with FCS (2%).

### Binding of *Sambucus nigra lectin* (SNA) and *Maackia amurensis lectin* (MAL) with parasites

Presence of linkage-specific Sias on different *L*. *donovani* strains was explored as described earlier [9, 23]. Parasite^+Sias^ and parasite^-Sias^ (1×10^7^/ml) were incubated with FITC conjugated SNA and MAA (5.0 μl, 5.0 μg/ml) for 1 hr at 4°C. Cells were washed in PBS and FITC positivity was acquired in flow cytometry (BD Biosciences) and by analyzing 10000 cells in CellQuestPro software.

### Binding of FITC-parasites with mammalian cells

Binding of FITC-parasite^+Sias^ with CHO cells was tested as described before [22]. CHO-siglec-1, CHO-siglec-3, CHO-siglec-5, CHO-siglec-7, CHO-siglec-9 and CHO-siglec-10 along with CHO-WT cells were either left untreated or blocked with specific anti-siglec-antibodies for 1 hr at 4°C. Cells were washed and incubated with parasite^+Sias^ and sialidase treated parasite^+Sias^ separately at 1:10 (CHO cells: parasite) ratio for 1 hr at 4°C. Unbound parasites were removed by washing and binding was measured by flow cytometry. Comparative binding of AG83^+Sias^, AG83^-Sias^ and UR6 with J774A.1 and THP-1 cells were evaluated similarly.

### Confocal microscopy

Sias-siglec interaction-mediated adhesion of FITC-parasite with J774A.1 was visualized by confocal microscopy [21]. Briefly, J774A.1 cells (2×10^4^) seeded on a glass coverslip grown for 48 hr in IMDM medium. One set of J774A.1 cells were prior blocked with anti-siglec-antibodies (anti-siglec-1, E and F) for 1 hr at 4°C. FITC-AG83/UR6 (sialidase-treated and untreated) was incubated with J774A.1 cells (anti-siglec antibody-treated/ untreated, 1:10 ratio) for 30 min at 4°C. Unbound parasites were washed with PBS. Subsequently, these macrophages were labeled with phycoerythrin (PE) conjugated anti-CD14 antibody for 30 min at 4°C. Cells were washed, fixed in paraformaldehyde and mounted in mounting media containing DAPI to stain nucleus. Images were acquired by Andor Spinning Disc Confocal microscope (Belfast, U.K.) with a 60×/1.42 NA oil immersion objective.

### Flow cytometric analysis of phagocytosis

Phagocytosis was performed as stated earlier with modifications [24]. J774A.1 cells (1×10^6^) were preincubated with anti-murine-siglec-1(anti-Sn) for 1 hr at 4°C in PBS-BSA (2%) or left untreated. These cells were infected with FITC-AG83^+Sias^ and AG83^-Sias^ (1: 10 ratio) for 2 hr at 37°C, which is the physiological temperature for phagocytosis. Unbound parasites were removed and the fluorescence intensity coming from extracellular non-phagocytosed FITC-AG83 was quenched by trypan blue treatment. Trypan blue quench the fluorescence of FITC by shifting the fluorescence from Fl1 channel to Fl3 channel. So, no signal from the surface of the cell will be detected in Fl1. Only the intracellular FITC signal will be detected in the Fl1. Therefore, visible intracellular fluorescence intensity was only due to phagocytosed parasites. This fluorescence was determined by flow cytometry.

### Measurement of extracellular phagocytosis

Siglec1-dependent phagocytosis of AG83^+Sias^ promastigote was also visualized by confocal microscopy [21]. J774A.1 cells were left untreated or treated with anti-murine-siglec-1, were infected with FITC-AG83^+Sias^ (sialidase-treated/untreated) for 2 hr at 37°C. Unbound parasites were washed; extracellular fluorescence was quenched by trypan blue and processed similarly as described in the previous section of confocal microscopy.

### Quantitation of nitric oxide (NO)

Nitric oxide was measured in cultured supernatant by Griess reaction as stated earlier [25]. Briefly, J774A.1 cells (1×10^6^/well) were infected with AG83^+Sias^ or AG83^-Sias^ or UR6 at 1:10 ratio for 4 hr in a six well plate. After the infection, unbound parasites were washed out and the cells were kept in desialylated FCS-containing medium for additional 20 hr. FCS was desialylated by treating with 2M propionic acid at 80°C for 2.5 hr followed by extensive dialysis. Culture supernatant was collected and accessed the accumulated nitrite.

### Detection of reactive oxygen species (ROS)

ROS generation was measured as described earlier [26]. Briefly, J774A.1 cells (1×10^6^) were either left uninfected or infected with AG83^+Sias^ or AG83^-Sias^ or UR6 for 1 hr. Unbound parasites were removed, macrophages were washed and stained with H_2_DCFDA (10 μM) for 30 min at 37°C. Cells were acquired in FACS similarly.

### Measurement of intracellular parasites by Giemsa staining

J774A.1 cells (2×10^4^) were grown on glass coverslip and left untreated or blocked with anti-murine siglec antibodies (anti-siglec-1,-E,-F) for 1 hr. They were infected with AG83^+Sias^ or AG83^-Sias^ or UR6 at 1: 10 ratio for 4 hr. Unbound parasites were removed. Infection was continued for additional 20 hr in desialylated serum-containing IMDM medium. The coverslip was methanol fixed and stained with Giemsa. Intracellular parasites were counted manually in light microscope (Ziess) at oil immersion (100×). The value was represented by amastigote number per 100 macrophages [27].

### Determination of secreted cytokines

Bone marrow derived macrophages (BMDM) were isolated as stated earlier [28]. BMDM (1×10^6^) were left either uninfected or infected with AG83^+Sias^ or AG83^-Sias^ or UR6 for 4 hr. Unbound parasites were removed and infection was allowed for additional 20 hr in desialylated serum containing medium in presence of lipopolysaccharides (LPS) (2.5 μg/ml) for secretion of Th2 or phorbol-12-myristate 13-acetate (PMA) (25 ng/ml)-ionomycin (1.0 μg/ml) for Th1 cytokines. Cell-free suspension was used for cytokines measurement by ELISA kit [29, 30].

### Genetic expression profiling by reverse transcriptase (RT) and real time PCR

J774A.1 cells were infected and stimulated similarly as stated in previous section and total mRNA was isolated by RNeasy Mini Kit as per manufacturer’s instructions. RNA (1.0 μg) was reverse transcribed to cDNA by Reverse Transcriptase kit. For RT-PCR analysis, the cDNA was amplified using specific primers targeting genes encoding for the cytokines namely IL-2, IL-4, IL-10, IFNγ, TGFβ, GAPDH and *iNOS* in a perkin-Elmer DNA thermal cycler. Primer sequences were listed in Table 1. The reaction product was separated in 1% agarose gel stained with ethidium bromide and image captured in BioRad Gel Documentation system [23]. Densitometric score was measured by ImageJ software.

**Table 1:** Primer sequences.

Primer		Primer sequence (5'-3')	Primer Length	Tm(°C)
IFNγ	Sense Primer	CAAGTGGCATAGATGTGGAAGA	22	54.7
	Antisense Primer	GTGGGTTGTTGACCTCAAACTT	22	55.7
IL-2	Sense Primer	CAGGATGGAGAATTACAGGA	20	51.2
	Antisense Primer	GTTATTGAGGGCTTGTTGAG	20	51.2
IL-4	Sense Primer	CAACGAAGAACACCACAGAGAG	22	55.6
	Antisense Primer	GATGTGGACTTGGACTCATTCA	22	54.6
IL-10	Sense Primer	CTAACGGAAACAACTCCTTG	20	51.0
	Antisense Primer	GAAAGGACACCATAGCAAAG	20	51.2
TGFβ	Sense Primer	CCCTAGATTTTGACTTGCAC	20	51.2
	Antisense Primer	GCCCAGTCACTAAGACTCTG	20	54.7
*iNOS*	Sense Primer	ACCTGAAAGAGGAAAAGGAC	20	52.2
	Antisense Primer	GGAGCCATAATACTGGTTGA	20	51.7
GAPDH	Sense Primer	GGAAGGACTCATGACCACAG	20	55.0
	Antisense Primer	GTCAGGTCCACCACTGACAC	20	57.7
18s rRNA	Sense Primer	GCTCATTAAATCAGTTATGG	20	46.0
	Antisense Primer	ACTACCATCGAAAGTTGATA	20	46.0
β-actin	Sense Primer	TGCTATGTTGCTCTAGACTT	20	48.0
	Antisense Primer	ATAGAGGTCTTTACGGATGT	20	48.0

Real time PCR analysis was performed by mixing cDNA with 2× SYBR green master mix on Roche Applied Science light cycler 480.0 instrument using software version 1.5.0. Relative quantification of each target gene were normalized to two housekeeping genes (18s rRNA and α β-actin) and expressed as a fold change compared with uninfected control using the comparative cycle threshold (CT) method [31].

### Immunoblot and immunoprecipitation analysis

J774A.1 (1×10^6^) were left either uninfected or infected with AG83^+Sias^ or AG83^-Sias^ or UR6 at 1:10 ratio (macrophage: parasite) for 4 hr, washed and cell lysate was prepared. Equal amount of protein from each sample was separated in SDS-PAGE (10%) and transferred into nitrocellulose membrane. Membrane was blocked with tris buffer saline (TBS)- BSA (2%) and incubated with specific primary antibody (1:1000 dilutions) overnight at 4°C followed by adding species specific HRP-conjugated secondary antibody (1:1000 dilutions). The signal was detected by West-pico ECL system (Pierce, Thermo Scientific, USA) as described earlier [32].

For the immunoprecipitation assay, J774A.1 cells were infected with AG83^+Sias^ and AG83^-Sias^ for 2 hr at 37°C. They were processed similarly as stated in the above paragraph. Cell lysate was incubated with anti-SHP-1 antibody (1:100) overnight at 4°C. SHP-1 bound complex was pulled down with Sepharose-4B-protein-A. After washing, the complex was resolved by SDS-PAGE (10%), transferred to nitrocellulose membrane, incubated with anti-SHP-1 and anti-siglec-E antibodies and processed for ECL [32]. Densitometric score was measured by ImageJ software.

### Subcellular fractionation

J774A.1 cells (2×10^6^/well) were left uninfected or infected with AG83^+Sias^ or AG83^-Sias^ or UR6 for 12 hr in 1:10 ratio in a six well plate. After infection, unbound parasites were removed. Cells were washed and fractionated into cytosol and nuclear portion by NE-PER kit according to manufacturer’s protocol. Briefly, cell pellet was suspended in cytosolic extraction reagent, vortexed, spin down and the supernatant was collected as cytosolic fraction. The residual mass was suspended in nuclear extraction reagent, vortexed rigorously, spin down and the supernatant was used as nuclear fraction [32]. Both the fractions were separated in SDS-PAGE gel, transferred and processed for western blot as described in previous section and developed similarly.

### Knockdown of siglec-E by siRNA treatment

Short interfering RNA (siRNA) was designed against murine siglec-E and transiently transfected in pre-seeded J774A.1 (1×10^6^) cells using lipofectamine and plus reagent according to the manufacturer’s instructions. Cells were incubated with siRNA for 6 hr. Transfection reagent was removed and further cultured in complete medium for 18 hr. Confirmation of siRNA-mediated knockdown of target gene was evaluated by western blotting [26].

Transfected and untransfected cells (absence of siRNA) were infected with AG83^+Sias^. Status of several effector molecules such as ROS, NO, cytokines and various signaling molecules were determined similarly as stated in the previous sections.

### Statistical analysis

The data were the mean value derived from at least three independent experiments. Statistical analysis was performed using the two-tailed Student’s t test. Error-bars represent mean ± SD from three independent experiments. Significant differences were set at * p≤ 0.05, ** p≤ 0.01, *** p ≤ 0.001 and analyzed by GraphPad Prism version 5.01.

## Results

### Sialic acids containing promastigotes exhibited enhanced siglec-1 and -5-mediated binding with macrophages

Although the differential distribution of Sias along with 9-*O*-acetylated Sias in virulent (AG83 and GE1) and avirulent (UR6) promastigotes of *L*. *donovani* were earlier reported [11], the presence of α2–3 and α2–6 linked Sias was further evaluated on the surface of different virulent SAG sensitive (AG83 Dd8 and 2001) and SAG resistant (39 and GE1) typed/untyped strains along with an avirulent UR6 promastigotes. The comparative bindings of FITC-SNA and FITC-MAL were tested in all the strains by flow cytometry. Promastigotes (parasite^+Sias^) except the strain 39 showed significantly higher (p≤ 0.05) SNA binding than MAL suggesting the predominance of α2–6 linkage on the surface of *L*. *donovani*. Sialidase-treated promastigotes (parasite^-Sias^) exhibited minimal binding (S1 Fig).

**Fig 1:**
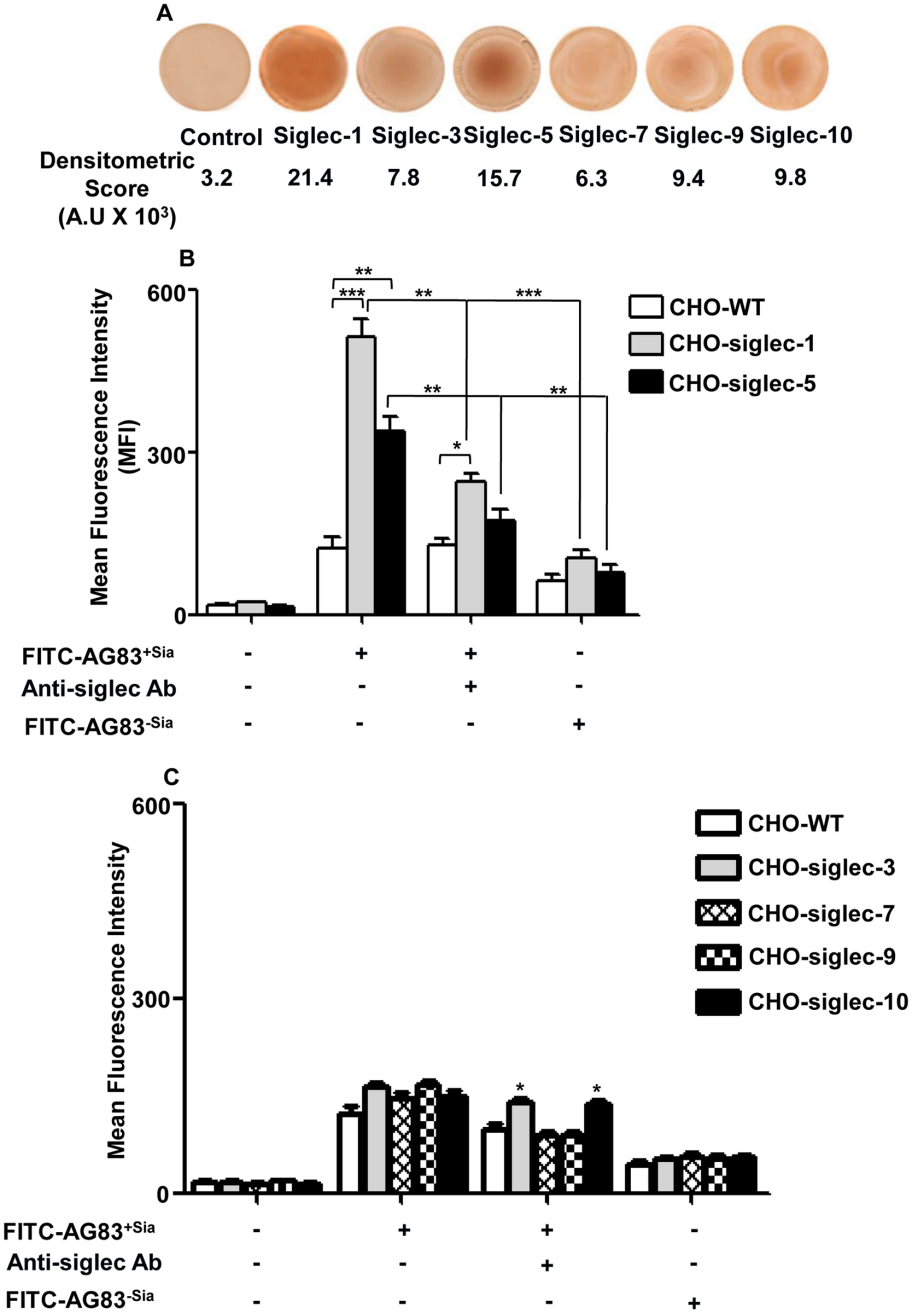
Identification of siglecs involved in binding of *Leishmania* promastigotes with the host cells. **(A)** Interaction between AG83^+Sias^ promastigotes and different soluble siglec-Fc chimeras (Siglec-1, 3, 5, 7, 9 and 10) was quantified in overlay dot blot. Promastigotes (1×10^7^), in PBS (50 μl) were soaked on nitrocellulose membrane. The air dried membrane was incubated with different soluble siglec-Fc chimeras followed by HRP-Protein-A and developed by DAB as described in materials and methods. **(B)** Sialic acids-siglec mediated binding of *L*. *donovani* (AG83^+Sias^) with CHO-siglec-1 and CHO-siglec-5 cells compared to CHO-WT. AG83^+Sias^ were left untreated or treated with esterase followed by sialidase (AG83^-Sias^) and labeled with FITC. In parallel, CHO-WT, CHO-siglec-1 and CHO-siglec-5 were untreated or incubated with specific anti-siglec antibodies. Subsequently, treated and untreated CHO cells were incubated with FITC-AG83^+Sias^/AG83^-Sias^ at 1: 10 ratio at 4°C and binding was measured by FACS. **(C)** Sias-siglec-mediated binding of AG83^+Sias^ with CHO-siglec-3, CHO-siglec-7, CHO-siglec-9 and CHO-siglec-10 compared to CHO-WT cells were measured after various treatments by FACS similarly as stated in Fig. 1B.

As virulent strain (AG83^+Sias^) showed higher linkage-specific Sias on the surface, here we selected this strain for the interaction with different siglecs. Initially, we compared the binding of different soluble siglecs (siglec-1,-3,-5,-7,-9,-10)-Fc chimera with AG83^+Sias^ by overlay dot blot (Fig 1A). Siglec-1-Fc chimera protein exhibited significantly (p ≤ 0.001) higher binding with the AG83^+Sias^; intensities being 21,450 ± 1130 followed by siglec-5-Fc (15,710 ± 790), than the control (3280 ± 265) containing only air-dried promastigotes. Other siglec-Fc chimera (siglec-3,-7,-9,-10) exhibited lower binding with AG83^+Sias^.

Binding of AG83^+Sias^ towards siglec-1 and-5 was further confirmed by using specific full length siglec-transfected CHO cell lines. Virulent strain (FITC-AG83^+Sias^) showed significantly enhanced binding with both CHO-siglec-1 (p ≤ 0.001) and CHO-siglec-5 (p ≤ 0.01) compared to CHO-WT as indicated by higher mean fluorescence intensity (MFI) value (Fig 1B). However blocking of siglec with specific anti-siglec antibody significantly (p ≤ 0.01) reduced the FITC-AG83^+Sias^ binding with CHO-siglec-1 and CHO-siglec-5 compared to untreated siglec-transfected CHO cells. In contrast, FITC-AG83^-Sias^ exhibited the significant reduction in binding with CHO-siglec-1 (p ≤ 0.001) and CHO-siglec-5 (p ≤ 0.01) compared to AG83^+Sias^. Conversely there was no significant enhancement in binding of FITC-AG83^+Sias^ with CHO- siglec-3, -7,-9,-10 cells compared to CHO-WT cells (Fig 1C).

Additionally, CHO-siglec-5 cells were treated with exogenous sialic acids (1.0 mM) prior to binding with parasite. There was significant (p ≤ 0.01) reduction in the binding of AG83^+Sias^ with pre-treated CHO-siglec-5 with MFI value being 2982 ± 378 compared to unblocked CHO-siglec 5 (4927 ± 422). However exogenous Sias treated CHO-siglec-1 cells showed no significant change in MFI value (8336 ± 680) compared to untreated control (9024 ± 805) under identical condition. This may be due to the elongated structure of siglec-1 that protruded way out from the cell surface and affinity towards linkage-specific sialic acids.

To confirm whether affinity of AG83^+Sias^ towards siglec-1 and -5 was a generalized phenomenon for *L*. *donovani*, this binding was further examined in other *L*. *donovani* strains also. Different *L*. *donovani* strains (except low Sias containing avirulent UR6) showed significantly (p≤ 0.01 & p≤ 0.001) enhanced binding with CHO-siglec-1 and CHO-siglec-5 compared to CHO-WT (S2 Fig).

### Sias-siglec interaction played a crucial role in binding of promastigotes with the macrophages

As AG83^+Sias^ promastigotes exhibited preferentially higher binding towards CHO-siglec-1 and CHO-siglec-5, next we examined such interaction using both murine macrophage (J774A.1) and human monocytic cells (THP-1) as they are the natural host of *Leishmania* parasite. Accordingly, the comparative binding of AG83^+Sias^/AG83^-Sias^ and UR6 with J774A.1/THP-1 cells was evaluated in the light of Sias-siglec interaction.

**Fig 2:**
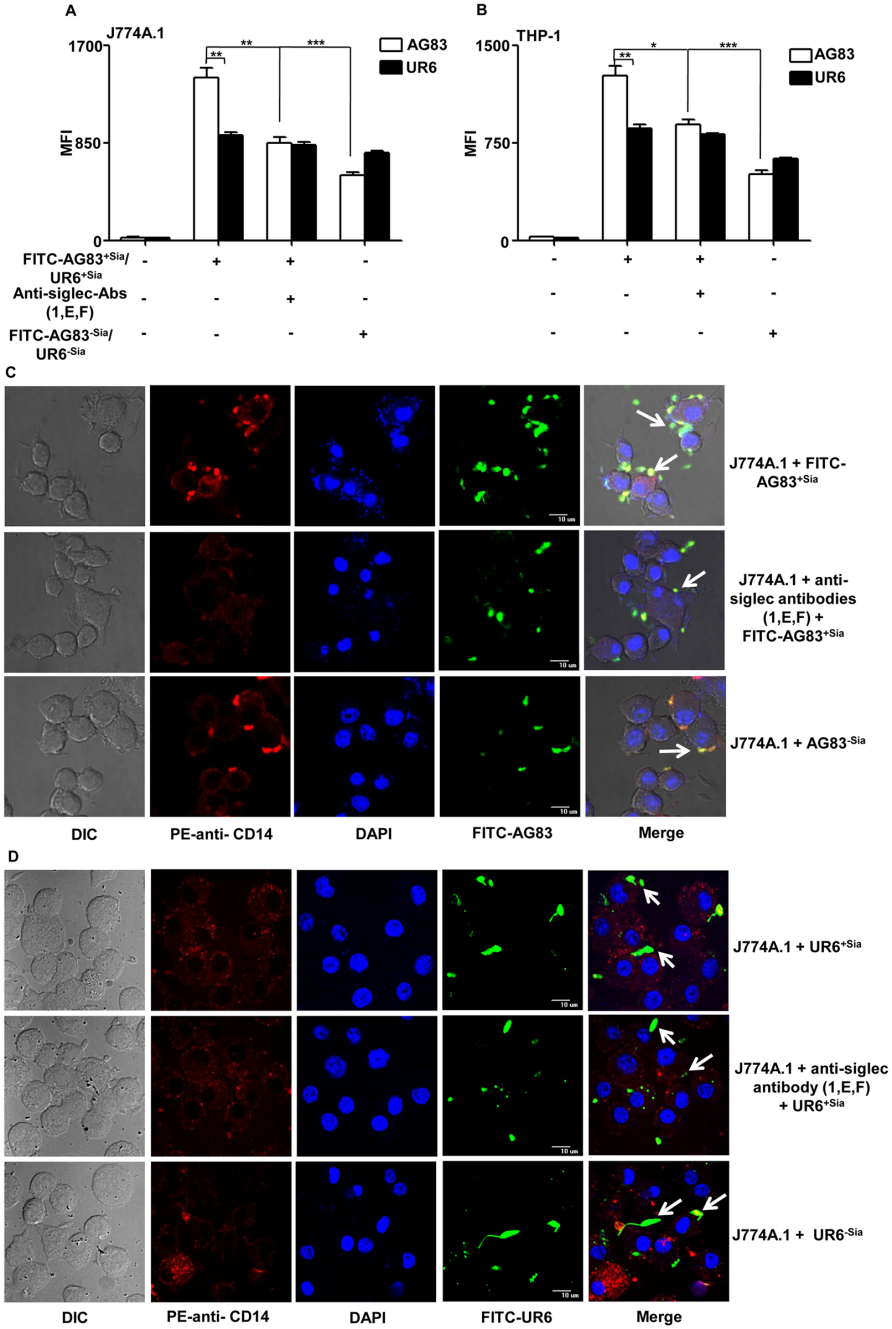
Comparison of Sias-siglec mediated binding between a virulent strain (AG83^+Sias^) and avirulent strain (UR6) with macrophages/monocytes. Sias-siglec mediated binding of AG83^+Sias^ and UR6 with J774A.1 **(A)** and THP-1 **(B)** cells were measured by flow cytometry. AG83^+Sias^ and UR6 were left untreated or treated with sialidase (AG83^-Sias^) and labeled with FITC. J774A.1/THP-1 cells were treated with cocktail of anti-siglec antibodies. Cocktail of antibodies were used as inhibitors due to the presence of many siglecs in variable amounts on J774A.1/THP-1 cells. Subsequently, FITC-AG83/UR6 promastigotes were incubated with untreated and treated J774A.1/THP-1 cells at 1:10 ratio and the binding was measured by gating the macrophages in FITC channel. Data were analyzed by CellQuestPro software. Data is represented as mean ± S.D from three independent experiments. *, p ≤ 0.05; **, p ≤ 0.01; ***, p ≤ 0.001. **(C)** J774A.1 cells were adhered on the glass coverslip, left untreated or treated with anti-siglec-1,-E,-F antibodies. FITC-AG83^+Sias^ or sialidase-treated (AG83^-Sias^) were incubated with treated and untreated J774A.1 cells at 4°C. Unbound promastigotes were removed; macrophages were stained with phycoerythrin (PE)-anti-CD14 and mounted with mounting media containing DAPI. Images were captured by Andor Spinning Disc Confocal microscope. Green colour represents the FITC-promastigote while red color indicated the surface of macrophages and DAPI stained nucleus were of blue color. **(D)** J774A.1 cells were adhered on the glass coverslip and treated in the same manner as in Fig. 2B. Cells were infected with FITC-UR6 promastigotes, stained and visualized by confocal microscopy as described in Fig. 2C.

FITC-AG83^+Sias^ exhibited significantly (p ≤ 0.001) enhanced binding with J774A.1 compared to UR6 as indicated by the higher MFI values (Fig 2A). In contrast, pretreated J774A.1 cells with cocktail anti-siglec antibodies against siglec-1,-E,-F followed by infection with AG83^+Sias^ exhibited extensively reduced (p ≤ 0.01) binding compared to untreated J774A.1 cells. Additionally, infection of J774A.1 with AG83^-Sias^ also showed decreased (p ≤ 0.001) binding compared to AG83^+Sias^. Interestingly binding of UR6 with macrophages remained unaltered in all these conditions. Similar trend of binding was observed when human monocytic cell line THP-1 was used (Fig 2B).

Sias-siglec-mediated interaction of FITC-AG83^+Sias^ promastigotes with J774A.1 was further visualized by confocal microscopy and shown as a representative image. Higher number of parasites attached to the macrophage surface was observed as indicated by green dots of FITC-AG83^+Sias^ on the PE-CD14 stained red colored periphery of the J774A.1 cells (Fig 2C). DAPI stained nucleus indicating intactness of the cell. Blocking of siglecs present on macrophages by specific anti-siglecs antibodies or removing sialic acids from parasites by sialidase treatment exhibited a fewer number of FITC-AG83 attached on the macrophage surface. As Sias present on UR6 are generally low, there were no apparent changes in their association with J774A.1 cells before and after inhibition of Sias-siglec interaction (Fig 2D).

### AG83^+ Sias^ exhibited enhanced siglec-1 mediated phagocytosis

**Fig 3:**
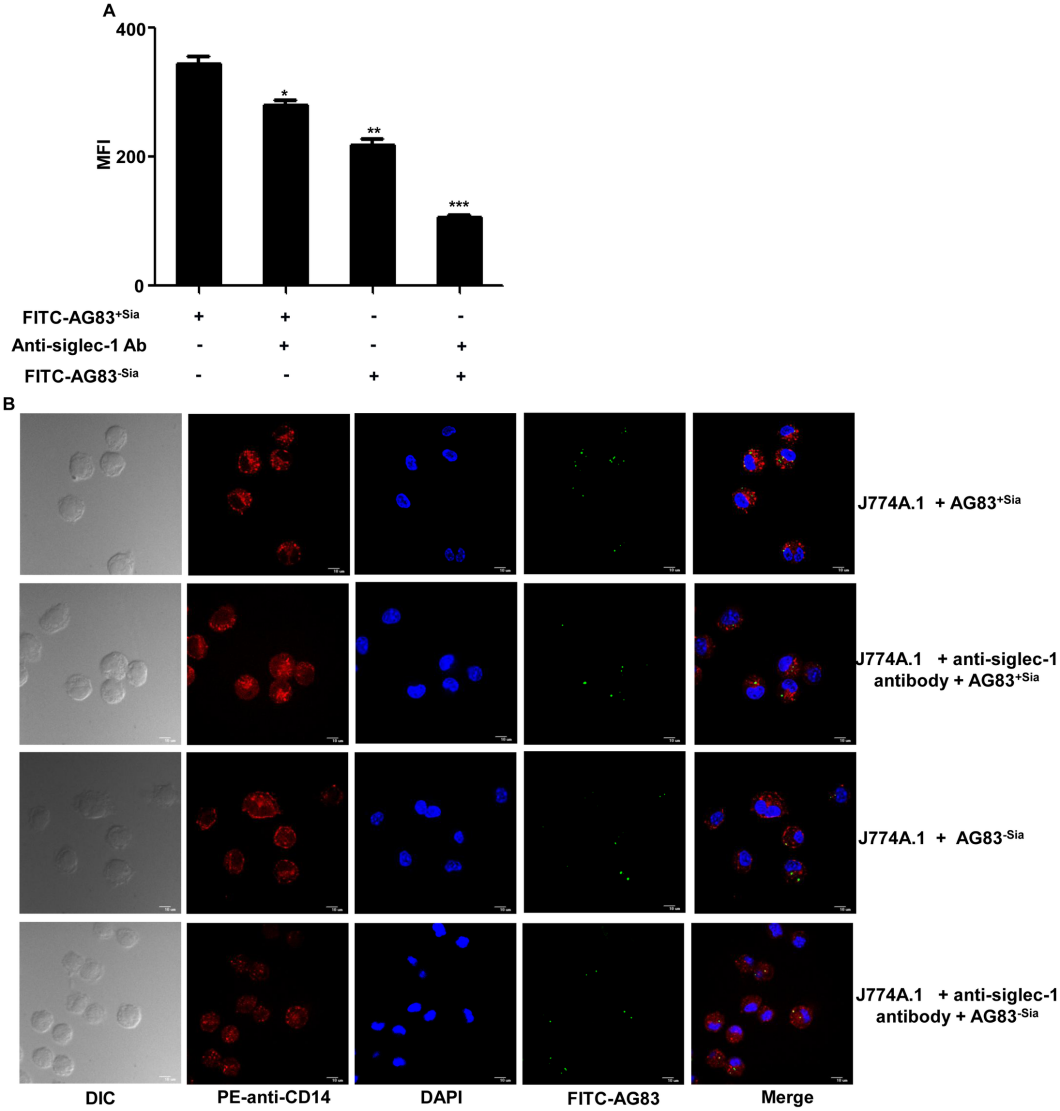
Siglec-1 mediated phagocytosis of promastigotes in macrophages. **(A)** Sias-siglec dependent phagocytosis was quantitated by flow cytometry. J774A.1 (1× 10^6^/well) cells were grown in a six well plate, either left untreated or blocked with anti-siglec-1 antibody (10 μg/ml) for 1 hr. Treated and untreated cells were then infected with FITC-AG83^+Sias^ and AG83^-Sias^ at 1:10 ratio for 2 hr at 37°C. Unbound parasite removed and extracellular fluorescence was quenched by trypan blue (0.02%) treatment. Cells were processed and visible fluorescence intensity was analyzed by flow cytometry which was due to phagocytosed parasites. **(B)** J774A.1 cells (2×10^4^) were adhered on glass coverslip for 48 hr. The cells were left untreated or blocked with anti-siglec-1 antibody for 1 hr at 4°C. They were infected with FITC-AG83^+Sias^ and FITC-AG83^-Sias^ promastigotes for phagocytosis as described in Fig. 3A. Infected macrophages were stained with PE-anti-CD14 antibody, fixed in paraformaldehyde and mounted in mounting media containing DAPI. The image was visualized in Andor Spinning Disc Confocal microscopy.

Phagocytosis is a physiological process by which cell uptake the extracellular component. Intracellular pathogens enter inside the cell through receptor-mediated phagocytosis. Here we quantitatively measured Sias-siglec-1 dependent uptake of promastigotes by flow cytometry (Fig 3A). This was also shown by confocal microscopy as a representative image (Fig 3B).

Sias-siglec mediated phagocytosis of AG83^+Sias^ showed higher MFI value which was significantly reduced (p ≤ 0.05 and ≤ 0.01) when this interaction was inhibited either by blocking of siglec-1 with specific antibody or removal of sialic acids from AG83^+Sias^ (AG83^-Sias^) before infection. This value was further reduced (p ≤ 0.001) when Sias-siglec-1 dependent phagocytosis was inhibited simultaneously both by blocking siglec-1 on macrophage and removing sialic acids from parasite.

A similar trend was observed in confocal microscopy also in which infected J774A.1 cells showed comparatively higher number of intracellular parasites as indicated by green dots than treated macrophages where Sias-siglec interaction was blocked, further confirming the role of sialic acids and siglec-1 in phagocytosis (Fig 3B).

### Sias-mediated interaction modulated the innate effector functions

**Fig 4:**
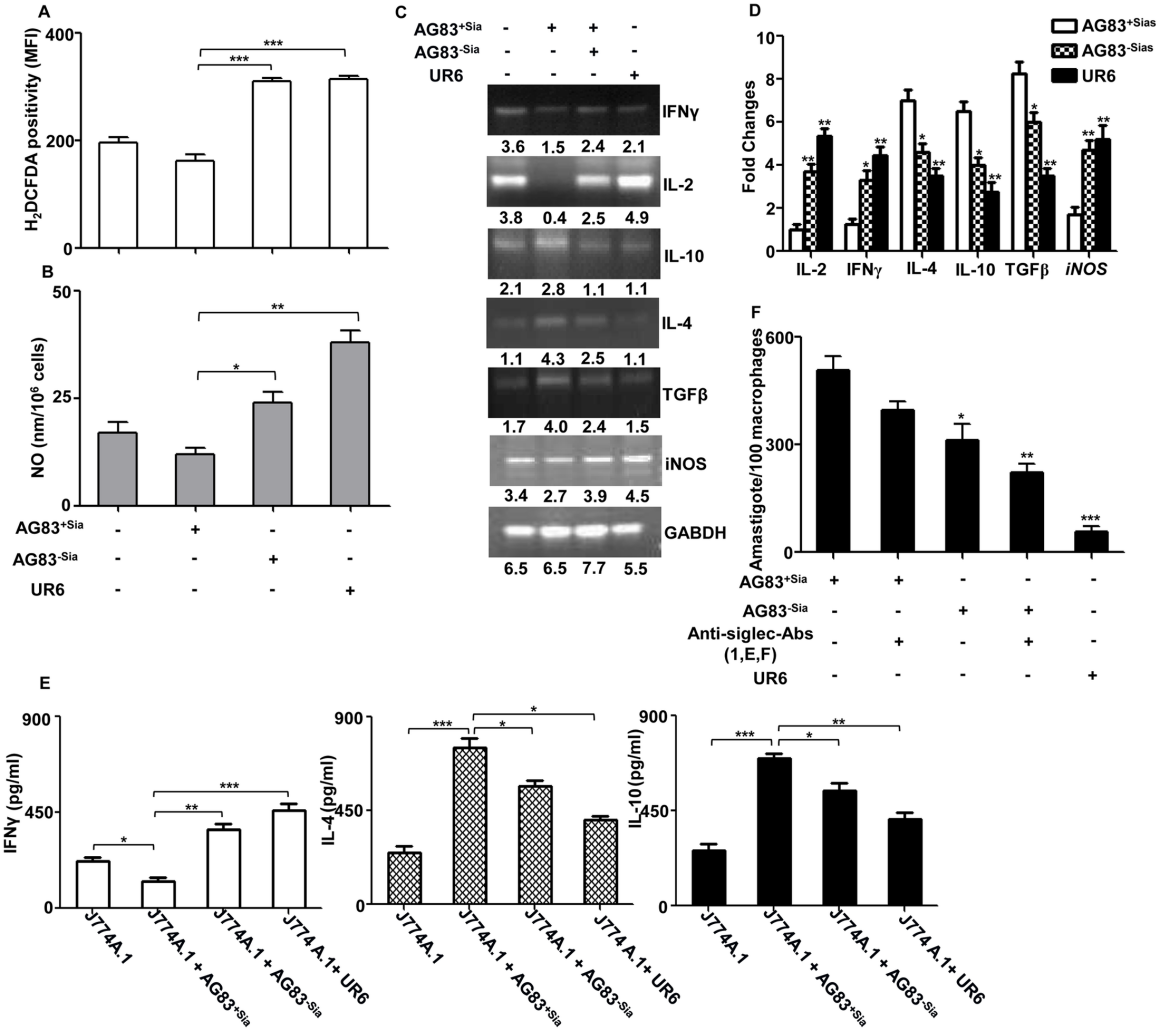
Sias-siglec interaction mediated suppression of innate immune response. **(A)** J774A.1 cells (1×10^6^/well) were left uninfected or infected with AG83^+Sias^ or sialidase-treated (AG83^-Sias^) or UR6 promastigotes in a six well plate at 1:10 ratio for 1 hr. Unbound parasites were removed and intracellular ROS production was measured by H_2_DCFDA staining by FACS. Data was analyzed by CellQuestPro software. **(B)** J774A.1 cells (1×10^6^/well) were kept uninfected or infected with AG83^+Sias^ or sialidase-treated (AG83^-Sias^) or UR6 similarly for 4 hr. Unbound parasites were washed and infection was allowed for additional 20 hr. The NO secretion was measured in the culture supernatant by Griess reaction. **(C)** J774A.1 cells (1×10^6^/well) infected with AG83^+Sias^ or AG83^-Sias^ or UR6 for 4 hr as stated in Fig. 4B. Infection was allowed for additional 20 hr in the presence of LPS (2.5 μg/ml) or PMA (25 ng/ml)-ionomycin (1.0 μg/ml) as stimulator for cytokines secretion. The total RNA was isolated by Quigen RNeasy Mini Kit. cDNA was prepared from RNA by reverse transcriptase Kit. Then the specific expression of the Th1 (IL-2, IFNγ) and Th2 (IL-4, IL-10, TGFβ) cytokine genes along with *iNOS* expression was measured in PCR by using specific primers as listed in Table 1. GAPDH was used as loading control throughout. **(D)** Total RNA was isolated from infected and uninfected J774A.1 cells and cDNA was prepared similarly as stated in Fig. 4C. The level of mRNA expression of Th1 and Th2 cytokines along with *iNOS* were quantified by real time PCR analysis. The mRNA level was normalized by 18s rRNA and the fold change was measured using uninfected cells as the control. Comparison was also made of AG83^+Sias^ infection with AG83^-Sias^ or UR6 infection. **(E)** Secreted cytokines were also measured in BMDM cells. BMDMs were isolated from the femur of healthy mice and macrophages were infected and stimulated by LPS or PMA-ionomycin as stated in Fig. 4C. The secreted cytokines (IL-4, IL-10 and IFNγ) in the culture supernatants were measured by respective ELISA kit as described in material and methods. **(F)** J774A.1 cells (1×10^4^) were adhered overnight on glass coverslip. Macrophages were left untreated or blocked with anti-siglec antibody cocktail (anti-siglec-1,-E,-F) for 1hr. Untreated and treated cells were infected with AG83^+Sias^ or AG83^-Sias^ or UR6 for 4 hr at 1:10 ratio. Unbound parasite removed and the infection was allowed for 20 hr. Macrophages were fixed in methanol and stained by Giemsa staining. Intracellular amastigotes were counted microscopically.

ROS and NO are two major effector molecules for the macrophage defense arsenal, which encounter the evading pathogens. As Sias-siglec interaction played a major role both in binding and phagocytosis of AG83^+sias^ in macrophages, we have extended our study to determine its effect in modulation of effector function. Due to the minimal presence of sialic acids on AG83^-Sias^ or UR6, J774A.1 cells were able to produce significantly higher (p ≤ 0.001) amount of ROS compared to infection with AG83^+sias^ as indicated by higher H_2_DCFDA positivity (Fig 4A).

Furthermore, during the AG83^+Sias^ infection, there was a reduction in the NO level in the culture supernatant indicating Sias-mediated interaction in modulation of innate effector function of the macrophages (Fig 4B). In contrast, infection with the AG83^-Sias^ or UR6, where Sias content is low, exhibited enhanced NO production (p≤ 0.05 & p≤ 0.01) indicating less infection.

### Sias-mediated interaction modulated the cytokine level in infected macrophages

*Leishmania* infection is generally associated with Th2 type of response *in vitro*. As genes ultimately regulate the expression of proteins through mRNA, so the actual status of any changes of cytokines and NO synthesizing gene *iNOS* during infection were evaluated at genetic level through reverse transcriptase (Fig 4C) as well as real time PCR (Fig 4D). Due to infection of macrophages with AG83^+Sias^, reduction of the genetic expression of Th1 cytokine genes (IFNγ and IL-2) and upregulation of Th2 cytokine genes (IL-4, IL-10 and TGFβ) were observed (Fig 4C). Removal of Sias from AG83 surface reversed the expression pattern of Th1/Th2 in the infected macrophages. This was further confirmed by infecting macrophages with avirulent UR6 strain. As expected, there was a significant reduction in expression of Th2 cytokines and upregulation of Th1 cytokines indicating the host protective immune response. The status of *iNOS* expression was also reduced upon infection of macrophages with AG83^+Sias^ compared to AG83^-Sias^ or UR6 infection.

This was further confirmed by evaluating mRNA transcript of different Th1 and Th2 cytokines by real time PCR. AG83^+Sias^ infected macrophages showed Th2 bias response, which was reversed to Th1 in AG83^-Sias^ and UR6-infected cells (Fig 4D). Th2 cytokines (IL-4, IL-10 and TGFβ) were upregulated by ~7.2, 6.3 and 8.3 folds respectively in macrophages infected with AG83^+Sias^, which were significantly (p≤ 0.05 and p≤ 0.01) down regulated to ~4.3, 4 and 6 folds respectively when AG83^-Sias^ was used. Infection with UR6 showed the further reduction in the Th2 cytokines to ~3.5, 2.8 and 3.5 folds respectively. On the other hand, Th1 cytokines (IL-2 and IFNγ) were significantly (p≤ 0.05) enhanced (~ 3.3–3.7 fold respectively) in AG83^-Sias^-infected macrophages and the level was further increased (~5.3 and 4.5 fold) when cells were infected with UR6 compared to AG83^+Sias^ (~ 1 and 1.3 fold respectively). Similarly *iNOS* level was only 1.7 fold increased in AG83^+Sias^ infected macrophages which was significantly (p≤ 0.05) upregulated to 4.8 fold and 5.2 fold during AG83^-Sias^ and UR6 infection. 18s rRNA was used as housekeeping gene for normalization. Similar trend was also observed for Th1/Th2 cytokines using β-actin as a normalizing housekeeping gene (S3 Fig).

Additionally, the status of a few key cytokines was determined in the culture supernatant. AG83^+Sias^-infected macrophages produced significantly (p≤ 0.05) less IFNγ level which was enhanced when AG83^-Sias^ or UR6 (p≤ 0.01 and p≤ 0.001) were used for infection (Fig 4E). In contrast, the increased levels of two immunosuppressive cytokines (IL-4 and IL-10) were observed during AG83^+Sias^ infection which was declined significantly (p≤ 0.05) during AG83^-Sias^ and UR6 infection.

### Sias-siglec interaction induced enhanced multiplication of intracellular amastigotes

Sias-siglec interaction played a significant contribution in enhanced phagocytosis and impairment of innate immune function in infected macrophages, thereby creating a perfect environment for multiplication of intracellular amastigotes. We have observed enhanced number of amastigotes in AG83^+Sias^ infected macrophages, which were significantly reduced (p≤ 0.05) when sialidase-treated AG83^+Sias^ was used for infection. Additionally, removal of sialic acids and blocking of siglecs together further significantly (p≤ 0.01) reduced the amastigote count confirming the role of Sias-siglec interaction in multiplication of intracellular parasites (Fig 4F). Low Sias containing avirulent strain (UR6) showed minimal level of infection than AG83^+Sias^.

### Sias-siglec interaction induced enhanced SHP-1 level in the host cell

**Fig 5:**
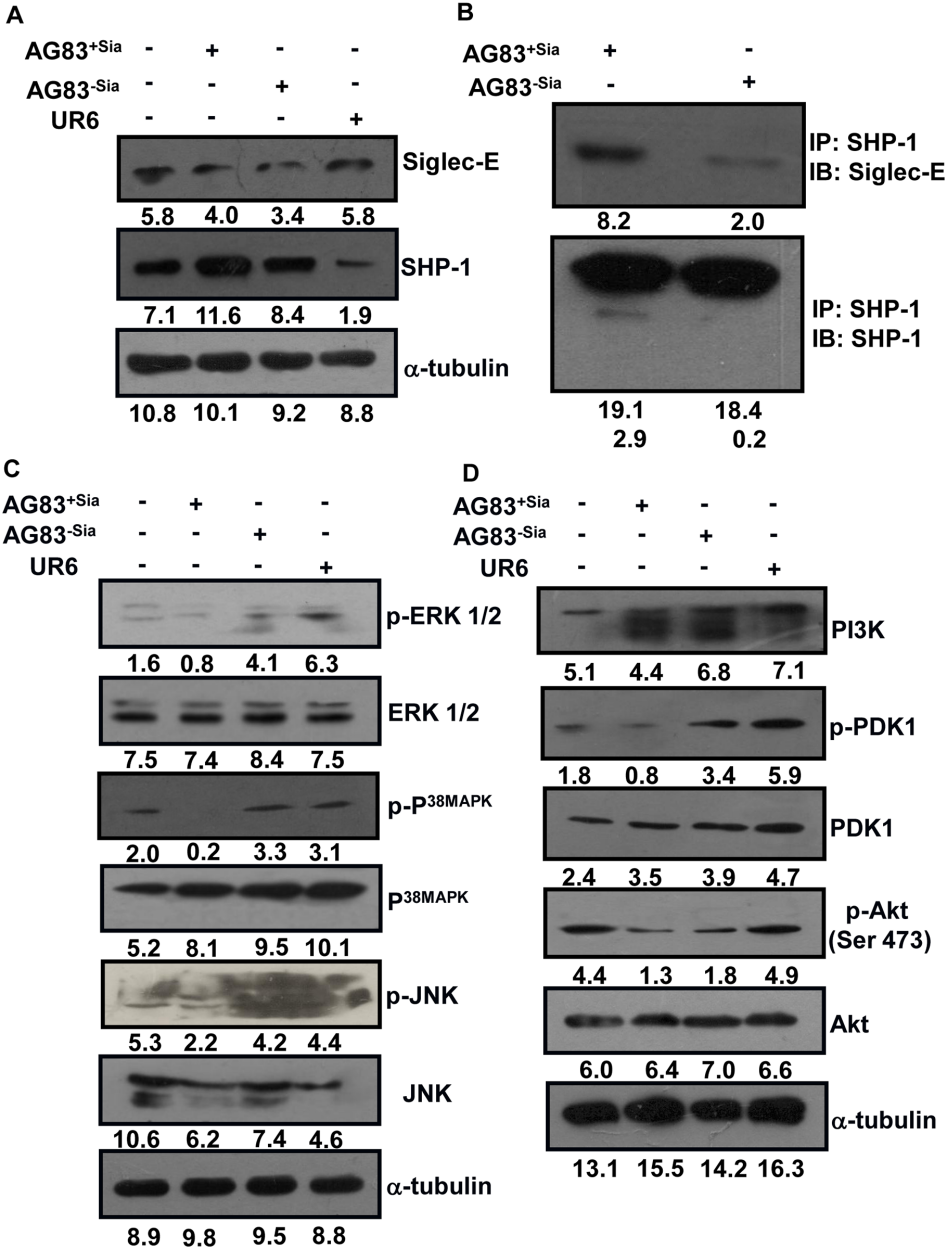
Sias-mediated modulation in the SHP-1 and downstream signaling pathways. **(A)** J774A.1 cells (1×10^6^/well) were uninfected or infected with AG83^+Sias^ or AG83^-Sias^ or UR6 at 1:10 ratio for 4 hr. Unbound parasites were removed. The cell lysate was prepared; proteins were quantified, separated in SDS-PAGE. They were transferred to nitrocellulose membrane and incubated overnight with anti-siglec-E and SHP-1 antibodies. α-tubulin was used as a control. The blots were incubated with respective secondary antibody and developed by ECL kit. Densitometric score mentioned below the each band was measured by ImageJ software. **(B)** Association of siglec-E and SHP-1 was measured by immunoprecipitation assay. J774A.1 cells were infected with AG83^+Sias^ or AG83^-Sias^ at 1:10 ratio for 2 hr. The cell lysate was incubated with anti-SHP-1 antibody overnight. The immunocomplex was pulled down by protein A-Sepharose 4B bead and run in SDS-PAGE. The proteins were transferred and processed similarly as in Fig. 5A. IP:Immunoprecipitation; IB: Immunoblot. **(C)** J774A.1 cells were infected and processed in similar manner as stated in Fig. 5A and incubated with anti-p-ERK-1/2, ERK-1/2, p-p38MAPK, p38MAPK, p-JNK, JNK and α-tubulin antibodies and developed. **(D)** Similarly, as stated in Fig. 5C, blots were also incubated with anti-PI3K, p-PDK1, PDK1, p-Akt (SER 473), Akt and α-tubulin antibodies and developed.

Next, we addressed the status of downstream molecular events that control the innate immune response for establishment of successful infection. Although siglec-1 and siglec-5 exhibited highest binding with AG83^+Sias^, only siglec-5 is capable to take part in signaling due to the presence of ITIM motif. Siglec-E, the murine counterpart of siglec-5 was quantified (Fig 5A). Western blot showed upregulation of SHP-1 in AG83^+Sias^ infected macrophages compared to AG83^-Sias^ and UR6-infected cells indicating the role of sialic acids in upregulation of this phosphatase. However, as expected we did not find any significant alteration in the siglec-E level in these infected cells. (Fig 5A).

To further confirm that upregulation of SHP-1 is specifically due to Sias-siglec mediated interaction, the association of SHP-1 with siglec-E was tested by immunoprecipitation (Fig 5B). The levels of both SHP-1 and siglec-E were higher in the SHP-1-siglec-E complex during AG83^+Sias^ infection compared to AG83^-Sias^ indicating enhanced association of siglec-E and SHP-1.

### Infection with AG83^+Sias^ inhibited the MAPKs and PI3K/Akt pathways

The correlation between sialic acids-mediated virulence factor in modulation of MAPKs and PI3K/Akt pathways during parasite infection was addressed further. Accordingly, the status of important molecules in these pathways was compared in J774A.1 cells infected with AG83^+Sias^, AG83^-Sias^ or UR6. Upon infection of J774A.1 with AG83^+Sias^, phosphorylation of ERK1/2, p38MAPKand JNK were suppressed compared to uninfected control (Fig 5C). In contrast, macrophages infected with AG83^-Sias^ restored the phosphorylation of these molecules. Similarly, low Sias-containing avirulent stain UR6 also failed to suppress the phosphorylation of the major immune activating MAPKs pathway molecules. However, total level of ERK1/2, p38MAPK and JNK remained constant upon infection.

Macrophages infected with AG83^+Sias^also induced the suppression of PI3K, p-PDK1 and p-Akt (Ser 473) in PI3K/Akt pathway (Fig 5D). Infection with AG83^-Sias^ showed the reversal of phosphorylation of these molecules indicating the role of Sias-mediated interaction by the parasite in deactivation of this pathway. Infection with UR6 also exhibited the similar trend of signaling in this pathway. However, total level of PDK1 and Akt remained constant upon infection.

### Sias-mediated interaction inhibited the translocation of a functional subunit of NF-κβ in the nucleus

**Fig 6:**
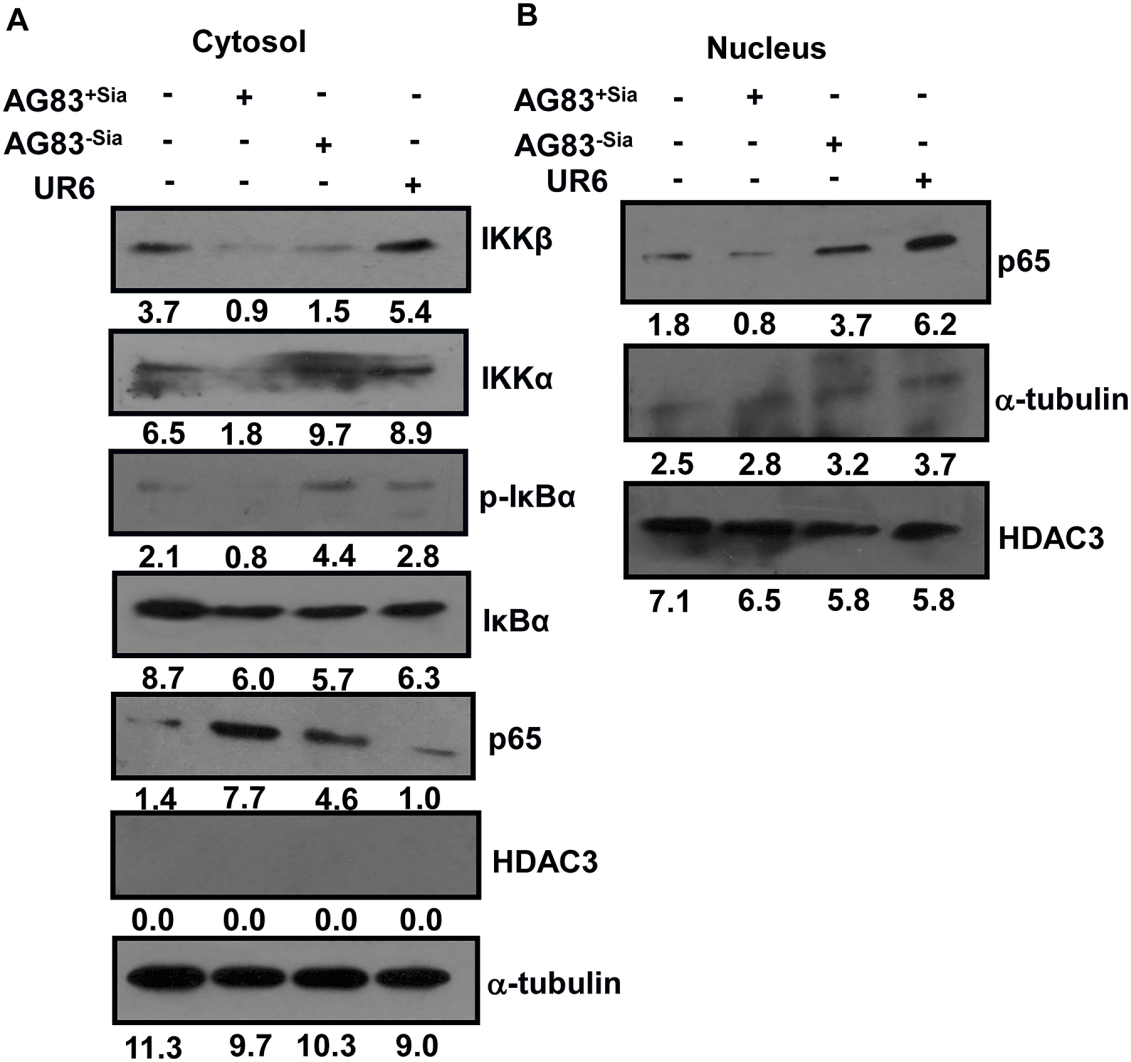
Sias-mediated interaction by *L*. *donovani* modulate a functional subunit of NF-κβ translocation in the nucleus. J774A.1 cells were uninfected or infected with AG83^+Sias^ or AG83^-Sias^ or UR6 at 1:10 for 12 hr, unbound parasites were removed. Cytosol **(A)** and Nuclear **(B)** fractions were separated as described in materials and methods and subjected to western blotting with indicated antibodies. Both α-tubulin and HDAC3 were used as loading controls for cytosol and nucleus respectively.

NF-κβ is a transcription factor translocate to the nucleus for transcription of proinflammatory genes. We have already observed the reduced macrophage effector function during AG83^+Sias^ infection. Therefore, the involvement of Sias-mediated interaction of *Leishmania* in the regulation of the translocation of a functional subunit of NF-κβ to the nucleus was assessed further. AG83^+Sias^ infection reduced the expression of IKKβ and IKKα in the cytoplasmic fraction of macrophages (Fig 6A). Subsequently, IKKα and IKKβ-mediated phosphorylation status of inhibitory subunit IκBα were also suppressed, indicating the prevention of autodegredation of this protein of NF-κβ complex in the cytosol. So, functional subunit p65 could not set free to translocate into the nucleus and thus accumulated more in the cytosolic fraction (Fig 6A). This reduces the chance of transcription of proinflammatory genes. In contrast, removal of Sias from virulent strain AG83^+Sias^ induced the expression of IKKβ and IKKα and, followed by IκBα phosphorylation-mediated degradation. This leads to enhanced translocation of p65 into the nucleus (Fig 6B). UR6 infection also showed similar kind of effect like AG83^-Sias^.

### Knocking down of siglec-E overturned the *Leishmania* induced regulation of macrophage effector function and signaling pathways

**Fig 7:**
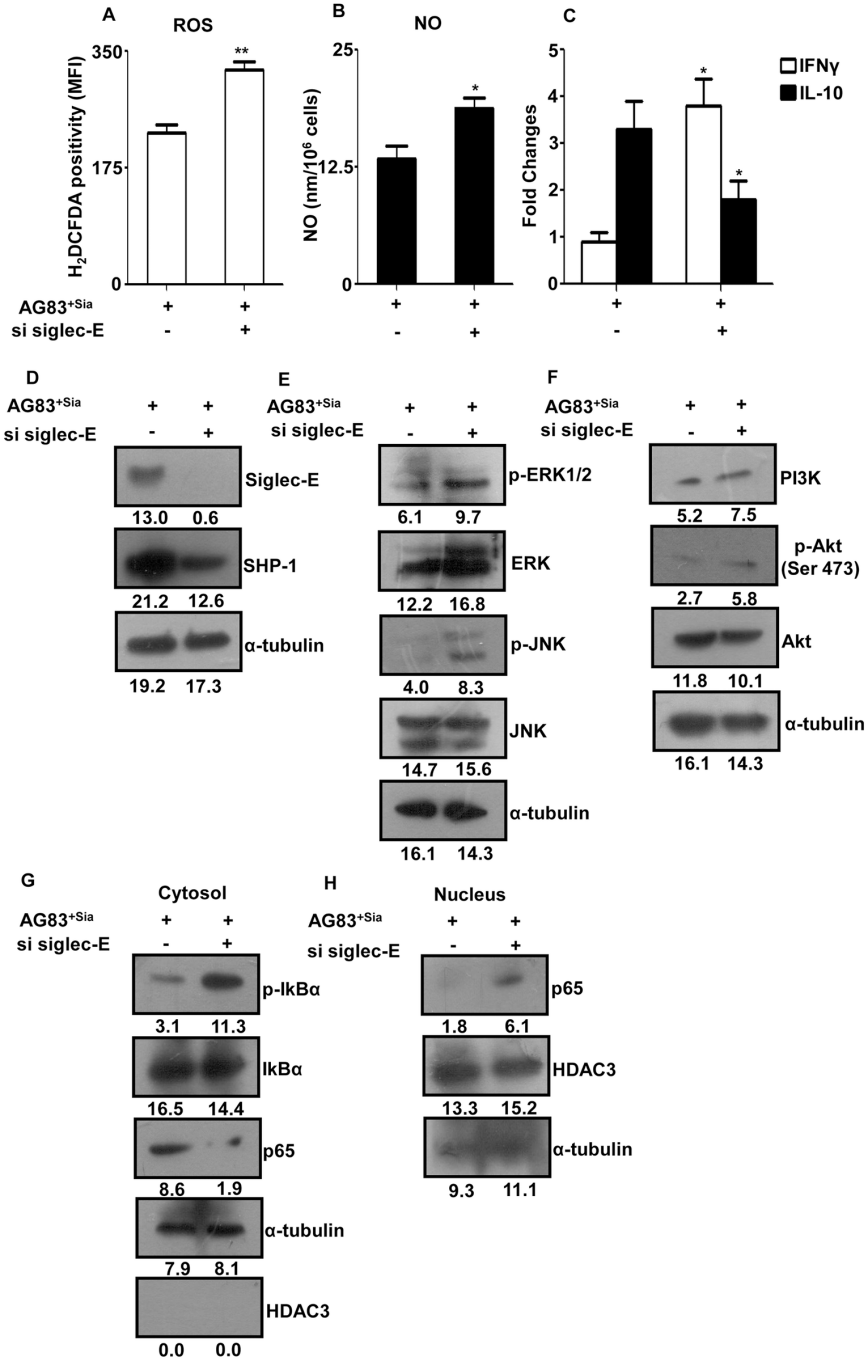
Siglec-E knockdown from macrophages modulated macrophage effector functions and downstream signaling molecules/pathways during *L*. *donovani* infection. **(A)** J774A.1 cells were either untransfected or transfected with siSiglec-E and infected with AG83^+Sias^ for 1 hr. ROS generation was measured by H_2_DCFDA staining similarly as stated in Fig 4A. **(B)** J774A.1 cells were transfected with siSiglec-E and infected with AG83^+Sias^ overnight. The culture supernatant was collected and nitric oxide was measured by Griess reaction as stated in Fig 4B. **(C)** Siglec-E depleted and control J774A.1 cells were infected with AG83^+Sias^ and the infection was allowed for 24 hr in presence of PMA-ionomycin or LPS as stated in Fig 4C. Total RNA was isolated and processed for quantification of IFNγ and IL-10 genes by real time PCR as stated in Fig 4D. **(D-F)** Untransfected or siSiglec-E-transfected J774A.1 cells were infected with AG83^+Sias^ for 4 hr. The cell lysate was prepared and western blotting of key signaling molecules were performed similarly as stated in Fig 5A. **(G-H)** J774A.1 cells were or transfected with siSiglec-E. Cytosol and nuclear fractions were separated and level of key signaling molecules were determined as stated in Fig 6.

Sialic acids-mediated interaction showed reduced macrophage effector functions and modulated downstream signaling molecules/pathway demanded to pin point the specific involvement of siglec (siglec-E) in these events. Accordingly, we transiently knocked down the expression of siglec-E by targeted siRNA in the macrophages. Infection of siglec-E depleted J774A.1 cells with AG83^+Sias^ showed enhanced (p≤ 0.05) ROS production with MFI value 322 ± 27 compared to untransfected cells (MFI 226 ± 31) (Fig 7A).

Similarly infection with AG83^+Sias^ in siglec-E-depleted J774A.1 cells significantly (p≤ 0.05) upregulated NO compared to untransfected macrophages confirming the importance of siglec in the suppression of macrophage effector function during *Leishmania* infection through Sias-siglec interaction (Fig 7B).

The status of key Th1 and Th2 cytokines during infection was also checked in siglec-E depleted macrophages. Significant (p≤ 0.05) upregulation of the IFNγ (~ 3.3 fold) was observed in transfected cells after infection compared to ~ 0.9 fold in untransfected macrophages. In contrast, IL-10 level was significantly (p≤ 0.05) down regulated (~ 1.8 fold) in siglec-E knockdown cells compared to ~ 3.9 fold in control cells due to the absence of Sias-siglec interaction during infection (Fig 7C).

Next, the level of phosphorylation of key signaling molecules in siglec-E depleted cells was checked to further confirm the importance of siglec in *L*. *donovani* infection through Sias-siglec interaction. Siglec-E depleted macrophages showed a complete absence of siglec-E molecule (Fig 7D). There was reduction in the SHP-1 level in these cells (Fig 7D). Other key signaling molecules in MAPK (Fig 7E) and PI3K/Akt (Fig 7F) pathway such as pERK, pJNK, PI3K and p-Akt were upregulated in AG83^+Sias^ infected siSiglec-E macrophages. Additionally, status of nuclear translocation of NK-κβ functional subunit p65 was also checked in siglec-E knockdown cells. We observed reduced presence of p65 in the cytosol (Fig 7G) along with enhanced phosphorylation of inhibitory molecule IκBα which leads to the enhance translocation of p65 in the nucleus (Fig 7H) indicating the potential role of siglec in Sias-siglec pathway utilized by *Leishmania* in modulation of signaling pathways in macrophages.

## Discussion

Tackling of visceral leishmaniasis is still challenging due to inadequacy of effective drug and development of widespread drug resistant strains [33]. Hence study the biology of host-parasite interaction is utmost important for identification of next generation drug target. Due to the unique location, sialic acids possess the strategic advantage to be a prime candidate for molecular interaction with host cells [34].

The most important findings of the present study include the demonstration of the involvement of sialic acids on virulent parasite for their binding with macrophages through siglecs, thereby establishing a new gate for the entry of this parasite. This Sias-siglec-mediated interaction of virulent parasite demonstrated its superior ability to subvert the innate immune response, enhanced multiplication of intracellular form of parasite in the host compared to low Sias-containing avirulent parasite. Current investigation also revealed a mechanistic approach to understand the specific role of Sias-siglec-1 in phagocytosis and involvement of Sias-siglec-E/5 in inducing suppression of MAPKs and PI3K/Akt pathways along with inhibition of translocation of a functional subunit of NF-κβ into the nucleus and thereby impairing the macrophage activation. The role of sialic acids and siglecs is thus established in ascertaining successful infection of *Leishmania donovani*.

Involvement of many *Leishmania* surface-associated glycoconjugates like lipophosphoglycan (LPG), glycoprotein 63 (gp63) in pathogenesis have been reported [35–41]. The presence of different derivatives of Sias both on promastigotes and amastigotes of *L*. *donovani* was identified in our laboratory [9, 10]. Our previous study also revealed the global distribution of Sias on different strains of *Leishmania* species [42]. However, the acquisition of Sias by *L*. *donovani* is still a matter of debate. Presence of any active metabolic enzyme (UDP-GlcNAc 2-epimerase) was not found in the parasite [9]. Whether this enzyme is presented as biologically non-functional remain to be investigated further. Whole genome sequencing of *L*. *donovani* revealed the two putative CMP-sialic acid transporters LDBPK_240360 (NC_018251.1) and LDBPK_240350 (NC_018251.1) located at chromosome number 24. This may be further linked with the presence of sialyltransferase activity in this parasite [43]. Based on the comparative study for the presence of linkage-specific sialic acids on different *L*. *donovani* strains including drug resistant, drug sensitive, typed, untyped and avirulent strain, a high sialic acids containing virulent strain AG83 was selected for this investigation.

There are many receptors present on macrophages for binding with mannose, fucose, fibronectin, Fcγ, complement etc. and actively take part in host-pathogen interactions [21, 44]. Siglecs, a unique class of membrane receptor, function as a negative regulator that prevents the over activation of immune cells. Our detailed investigation using siglec-transfected CHO cells along with macrophages/monocytes using five different strains conclusively confirmed that siglec-1 and -5 are two important candidate surface molecules served as receptors for virulent parasite binding. Specificity of sialic acids-siglec-mediated binding was confirmed by either removal of Sias or blocking siglecs. However, sialidase-treated parasite (AG83^-Sias^) didn’t show complete reduction in binding with macrophages which are considered to be the natural host for parasite. This may be due to the presence of number of other receptors available for binding with parasite on the surface of macrophage besides siglecs unlike CHO cells. Thus the presence of linkage-specific Sias on parasite surface and their capability to form the Sias-siglec interaction with macrophages could screen the virulent *vs*. avirulent parasite. Binding of biotinylated siglec-1,-2,-5 with *L*. *donovani* further confirmed our finding [43]. In contrast, other human pathogens such as *Pseudomonas aeruginosa*, Group B *Streptococcus* and *Campylobacter jejuni* exhibited specificity towards siglec-7/9; siglec-5/7/9 and siglec-1/7 respectively. This may also depends on the siglec-specific sialylated ligands present on the pathogen surface and their affinity towards specific siglecs [4, 45]. Therefore, siglecs are used by sialylated pathogens for their survival advantages [46].

After binding with the host cells, *Leishmania* promastigote initially phagocytosed by macrophages into the phagosome and subsequently fused with lysosome to form a phagolysosomal vacuole [47]. Thus phagocytosis is a prerequisite phenomenon in the life cycle of this pathogen. *Leishmania* undergo receptor-mediated phagocytosis and there are many candidate receptors that take part in this event [44]. Due to the elongated structure of siglec-1, it is extended way out from the cell surface making it a prime target for receptor-mediated phagocytosis by pathogens [7]. We have established siglec-1 as a new molecule that takes part in phagocytosis of AG83^+Sias^ during infection. Role of Sias-siglec-1 mediated phagocytosis was further confirmed as blocking of siglec-1 by antibody or removing Sias or combination treatment reduced the phagocytosed parasite inside the macrophages. Siglec-1 dependent phagocytosis was also reported in *Campylobactor jejuni* infection to the macrophages [48].

Impairment of innate immune system is an indispensable event for *L*. *donovani* to establish a successful infection. The immunosuppressive effect associated with leishmaniasis is exerted by the alteration of Th1/Th2 cytokine balance due to deactivation of macrophages induced effector molecules (ROS and NO) [49]. They were impaired during Sias-siglec mediated interaction of AG83^+Sias^ infection, indicating their direct involvement in immunosuppression. IFNγ and IL-4 are the key players involved both in VL and cutaneous leishmaniasis. However, IL-10 emerged as a most potent immunosuppressive cytokine in VL pathogenesis [50]. As cytokine secretion is more prolific in the primary macrophages, the secreted cytokines were measured in BMDM also. Sias-siglec mediated interaction of AG83^+Sias^ showed Th2 dominant (IL-10, IL-4 and TGFβ) cytokine response. All these events resulted in the enhanced intracellular amastigote replication. Thus impairment of this specific Sias-siglec interaction either by removal of Sias from parasite or silencing siglec from macrophages resulted in the reversal of immune response leading to reduction of parasite burden.

*L*. *donovani* engages a negative regulator SHP-1 during their infection to the macrophages for inactivation of the defense mechanism [51]. It is important to mention here that specific receptor present on macrophages as well as the molecular determinant on parasite which are directly involved in SHP-1 induction was not well documented. Present study conclusively demonstrated Sias-siglec-mediated interaction plays an important role in the upregulation of SHP-1 molecule and association between SHP-1 and siglec-E during infection. The specificity of the involvement of this receptor-ligand interaction for SHP-1 upregulation was further confirmed by removal of Sias from parasite or depleting siglec-E from macrophages.

SHPs act as negative regulator of NO production and suppressing MAPKs and Akt signaling pathways [52]. An array of molecular determinants present on *Leishmania* is known to deactivate these pathways [53, 54]. Here we have established an alternative pathway (Sias-siglec-mediated interaction) utilized by the virulent parasite (AG83^+Sias^) for deactivation of MAPK and PI3K/Akt pathway in the infected macrophages. Thus, Sias-siglec interaction could be considered as another new avenue of host pathogen interaction that can suppress the downstream signaling leading to deactivation of macrophages.

MAPKs and Akt pathways transmit the signal to the nucleus through a well known transcription factor NF-κβ, which control an array of proinflammatory genes [55]. We have demonstrated inhibition of nuclear translocation of a functional component (p65 subunit) of NF-κβ in AG83^+Sias^-infected macrophage through Sias-siglec-mediated interaction. This was correlated with the reduced ROS, NO and Th1 cytokines production. Removal of Sias or depletion of siglec showed reversal of this effect, indicating Sias-siglec interaction is an important determinant for immune suppression. A recent study on Group B *Streptococcus* also exhibited similar kind of distortion of innate immune response due to the sialic acids-mediated recruitment of siglec-E [24].

Our study conclusively ascertains that the Sias-siglec mediated interactions play a significant role in the binding and phagocytosis of virulent parasites with macrophages through siglec-1. This important molecular determinant (Sias) is essentially responsible for subversion of innate immune function by modulation of downstream signaling pathways favoring the multiplication of intracellular parasite in the hostile environment of host through siglec-E/5 (S4 Fig). Taken together, this in-depth study paved a novel horizon of host pathogen interaction, utilized by sialylated pathogens for their own survival advantages. Such finding may also help to design newer drug target. However, this needs further in-depth investigation.

## Supporting Information

S1 Fig Presence of α2–3 and α2–6 linked Sias on different *L*. *donovani* strains. Six different *L*. *donovani* strains as mentioned in materials and methods, were grown in 10% FCS containing medium was designated as parasite^+sias^ and sialidase (along with esterase) treatment of this parasite^+Sias^ was designated as parasite^-Sias^. Parasite^+Sias^ and parasite^-Sias^ were incubated with FITC-SNA (specific for α2–6 linked Sias) and MAA (specific for α2–3 linked Sias) and the binding was measured by flow cytometry.
(TIF)

S2 Fig Binding of different *L*. *donovani* strains with CHO-WT, CHO-siglec-1 and CHO-siglec-5. Binding of FITC-parasite^+Sia^ of five different *L*. *donovani* strains were incubated with different types of CHO-siglecs cells at 1: 10 ratio and binding was measured by FACS.
(TIF)

S3 Fig Real time PCR analysis of cytokines. Total RNA was isolated from control and infected macrophages and differential expression of mRNA of Th1 and Th2 cytokines were quantified similarly as stated in Fig 4D, using β-actin as housekeeping gene for normalization.
(TIF)

S4 Fig A schematic diagram of Sias-siglec interaction mediated phagocytosis and immunosuppression during *Leishmania* infection. (TIF)
