# Supplementary figures and images for: Leishmania donovani Utilize Sialic Acids for Binding and Phagocytosis in the Macrophages through Selective Utilization of Siglecs and Impair the Innate Immune Arm
Source: PLoS Negl Trop Dis. 2016 Aug 5;10(8):e0004904. doi: 10.1371/journal.pntd.0004904 (PMC4975436; doi:10.1371/journal.pntd.0004904)

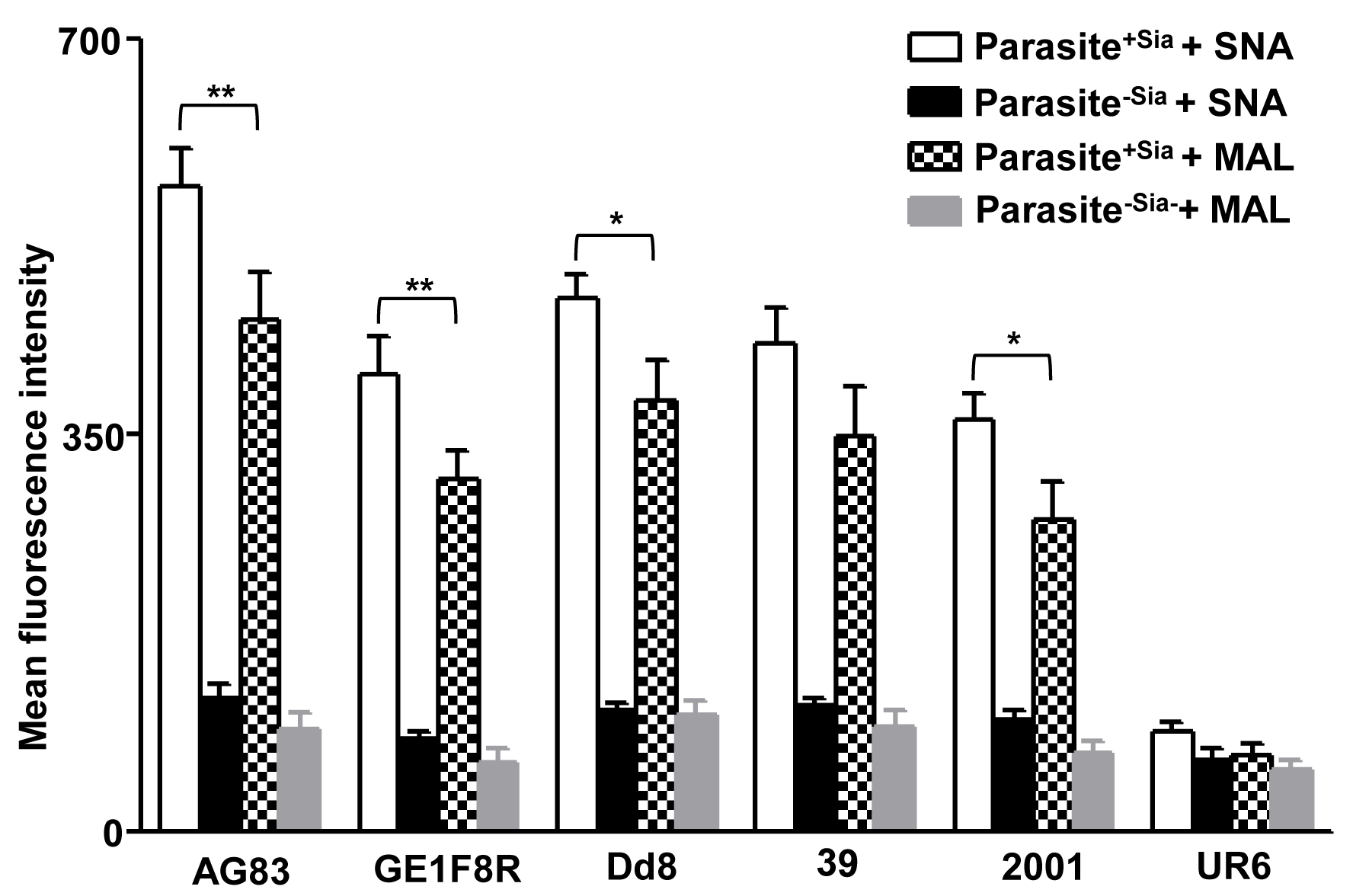

Supplement: S1 Fig — Six different L. donovani strains as mentioned in materials and methods, were grown in 10% FCS containing medium was designated as parasite+sias and sialidase (along with esterase) treatment of this parasite+Sias was designated as parasite-Sias. Parasite+Sias and parasite-Sias were incubated with FITC-SNA (specific for α2–6 linked Sias) and MAA (specific for α2–3 linked Sias) and the binding was measured by flow cytometry. (TIF) [file pntd.0004904.s001.tif]

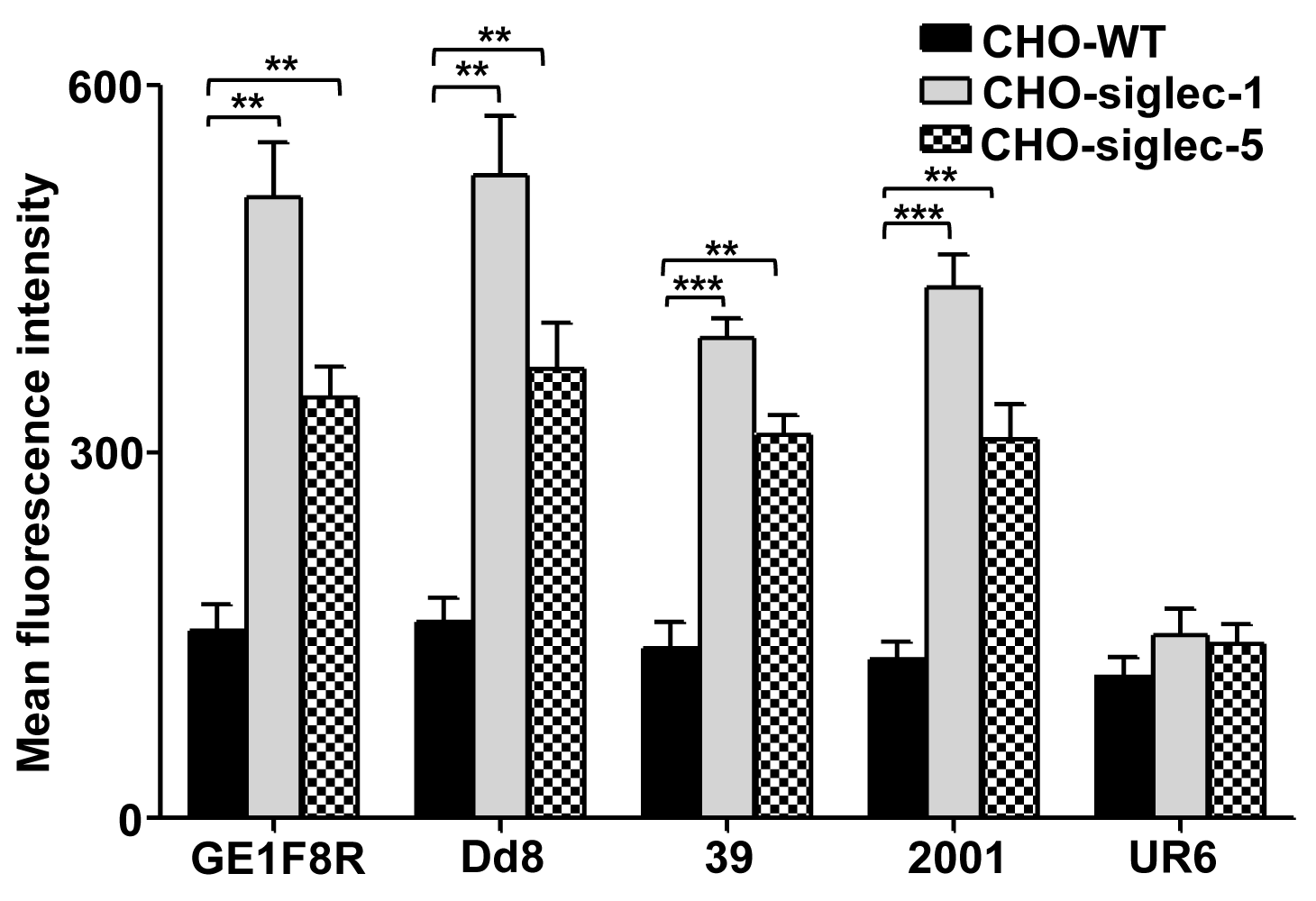

Supplement: S2 Fig — Binding of FITC-parasite+Sia of five different L. donovani strains were incubated with different types of CHO-siglecs cells at 1: 10 ratio and binding was measured by FACS. (TIF) [file pntd.0004904.s002.tif]

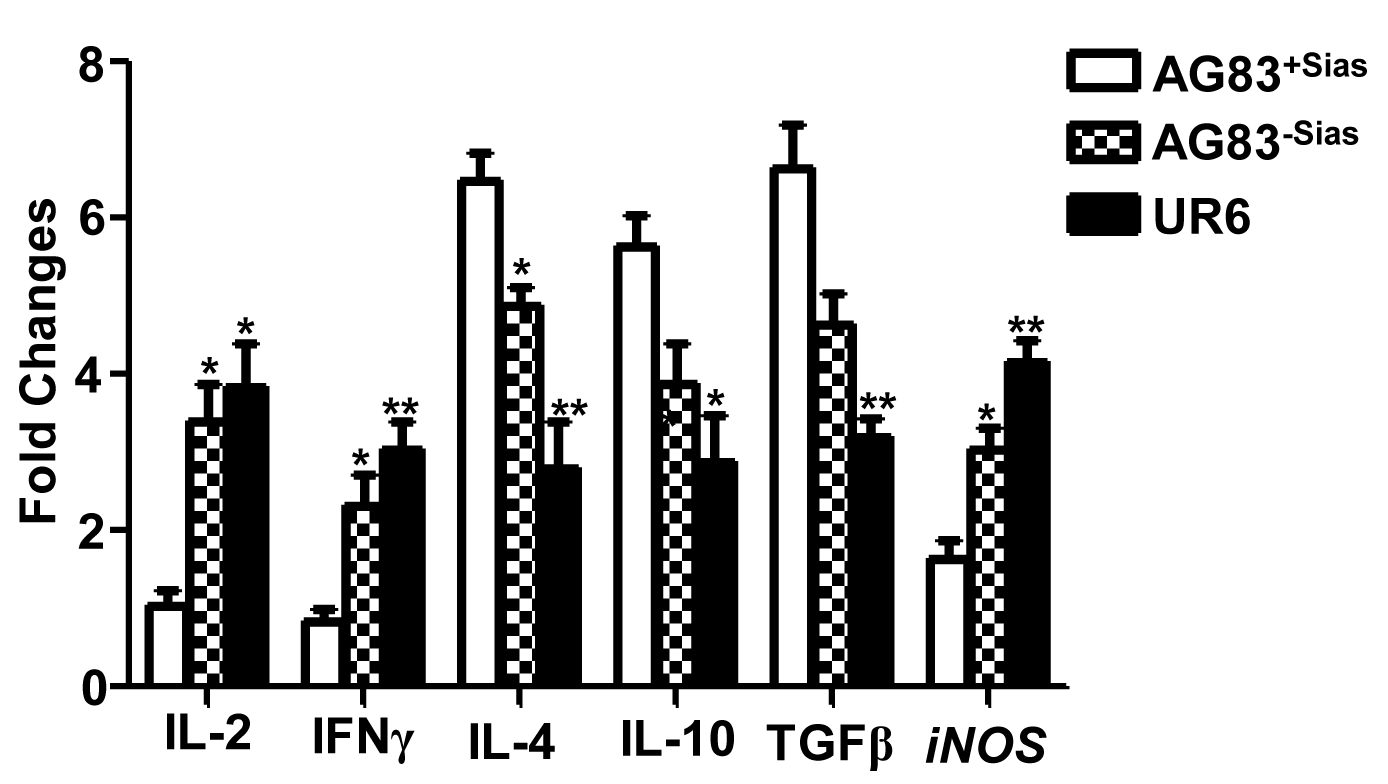

Supplement: S3 Fig — Total RNA was isolated from control and infected macrophages and differential expression of mRNA of Th1 and Th2 cytokines were quantified similarly as stated in Fig 4D, using β-actin as housekeeping gene for normalization. (TIF) [file pntd.0004904.s003.tif]

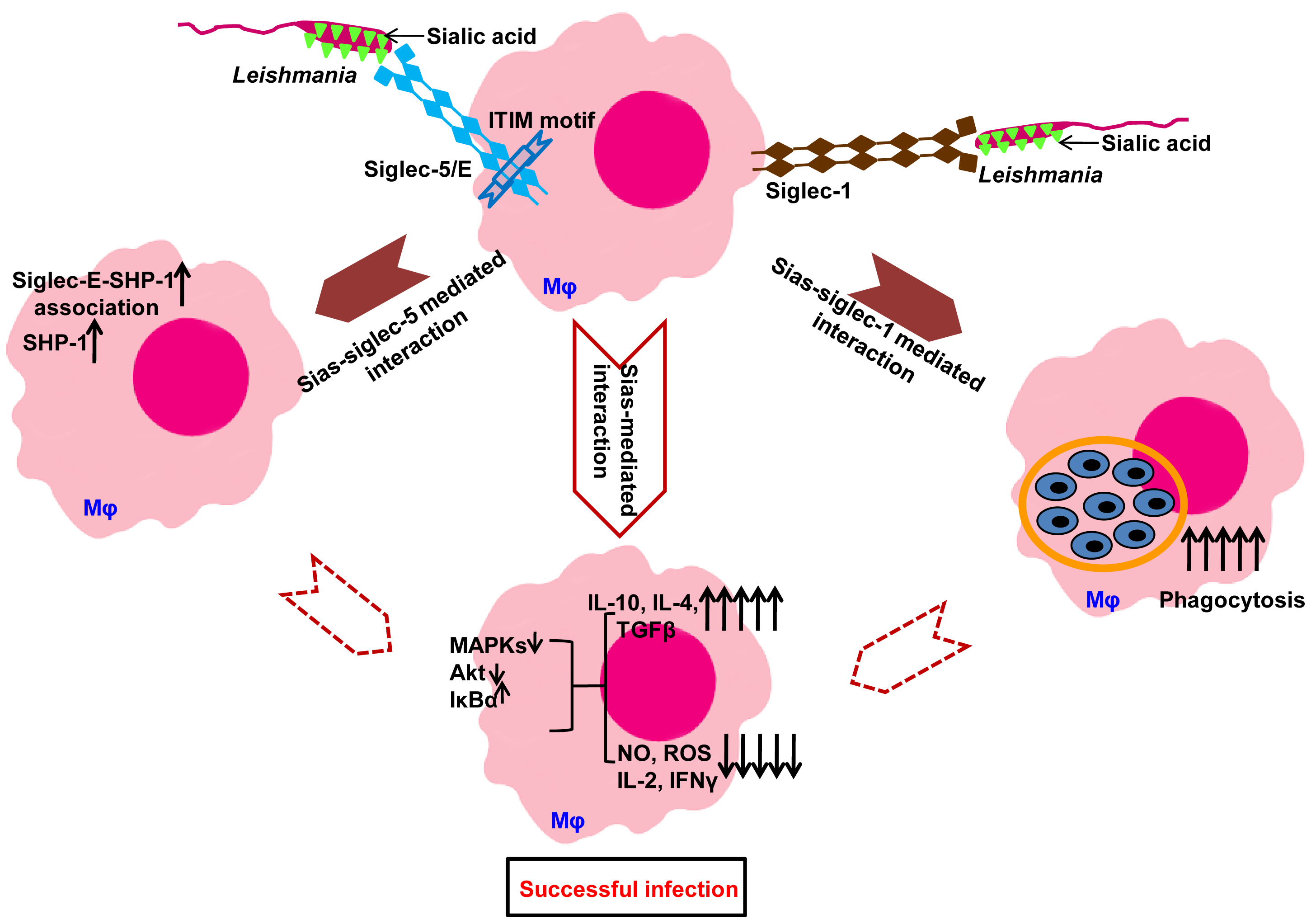

Supplement: S4 Fig — (TIF) [file pntd.0004904.s004.tif]
